# Interviews with Community Healthcare Registered Nurses in Norway: Examination Practices and Clinical Evaluation Processes

**DOI:** 10.1002/nop2.1045

**Published:** 2021-09-03

**Authors:** Geir V. Berg, Åshild Slettebø, Kjersti Johnsen, Aud Findal Dahl, Mariann Fossum

**Affiliations:** ^1^ Division Gjøvik and Lillehammer Faculty of Medicine and Health Sciences (MH) Department of Health Sciences in Gjøvik NTNU/Innlandet Hospital Trust Gjøvik Norway; ^2^ Centre for Caring Research – Southern Norway Faculty of Health and Sport Sciences University of Agder Kristiansand Norway

**Keywords:** clinical evaluation processes, community care, examination practices, individual interviews, Registered Nurse, thematic analysis

## Abstract

**Aim:**

This study describes the examination practices and clinical evaluation processes that Registered Nurses in Norway perform in the community healthcare sector.

**Design:**

A qualitative exploratory design.

**Methods:**

Twenty interviews were conducted with Registered Nurses employed in the community healthcare sector in Norway. The data were analysed using a thematic analysis.

**Results:**

We found four major themes: (1) evaluations are embedded in nurses’ daily work, (2) significance of a Registered Nurse's clinical competency, (3) different tasks require various roles and (4) access to resources and equipment. Registered Nurses possess several skills in a range of different examination techniques and clinical evaluation processes in the community healthcare sector. They perform complex assessments in their daily work and must rely on other healthcare professionals, facilities and equipment to provide high‐quality care. Ongoing education and training will enable Registered Nurses to complete accurate assessments in their community healthcare practice.

## 1 INTRODUCTION

Community healthcare nursing is “nursing that takes place outside of a hospital or institutional settings” (St John & Keleher, 2020, p. 4) and has become increasingly complex. The number of comprehensive and complex patient cases has continued to rise due to an ageing population characterized by a higher prevalence of chronic health conditions. The patient population that receives community‐based nursing care in Europe is characterized by multi‐morbidity, polypharmacy and/or cognitive impairment (Bing‐Jonsson et al., 2016). This combination leaves many elderly patients in frail mental and physical conditions with a high risk of adverse outcomes if their need for nursing care is not properly met.

## 2 BACKGROUND

To provide health care for patients in the community healthcare sector, each of whom is unique and has complex conditions, nurses must have advanced knowledge and skills (meaning that they possess relevant clinical competence that matches their patient groups and contexts) because this a key factor needed to achieve improved patient outcomes (Coffey et al., 2016). Their competency must be based on broad theoretical knowledge from nursing and other disciplines, as well as the ability to use their knowledge in clinical situations according to the patient's current status (St John & Keleher, 2020). Although the community healthcare sector is based on teamwork, community nurses often need to make solo decisions (Duner, 2013).

Knowing the patients well enough to plan care is fundamental for clinical decisions to be made (Gray et al., 2018). Gray et al. (2018) conducted an integrative review study on nurses’ clinical assessment and decision‐making—as well as the effects on patient outcomes—in hospital and home care. They argue that nurses play an important role in the assessment of patients, and that high competence in nursing assessment skills is vital in high‐quality decision‐making. Accurate decision‐making includes understanding patient situations and using standard protocols in nursing (Nibbelink & Brewer, 2018). Although intuition is crucial to nursing, nurses use both analysis and synthesis of intuition, in addition to objective parameters (Melin‐Johansson et al., 2017). In a recent study, Nilsen et al. (2019) found that checklists should be customized for each patient to provide holistic nursing care to older people in home healthcare services. Leaders’ roles in implementing checklists were also emphasized. Standard protocols—such as clinical screening tools and checklists for assessing patient's needs and guide care planning—are essential to support evidence‐based care and improve patient outcomes (Burgers et al., 2020; Mathieson et al., 2019).

Efforts have been made to enhance the quality of community‐based health care (Mathieson et al., 2019). However, there are several reports of inadequate care and adverse events (Phelan et al., 2018; Sworn & Booth, 2020). These indicate a need to address questions about health care and the required clinical skill levels of staff members. Having well‐educated staff members who can meet these patients’ needs is essential (Bing‐Jonsson et al., 2016 p.2). Bing‐Jonsson et al. (2016) conducted a cross‐sectional survey of 1,016 nursing staff members employed in nursing homes and home care services using the instrument, “Nursing Older People—Competence Evaluation Tool.” They found that the level of competence was insufficient in the areas of nursing measures, advanced procedure, and documentation. They recommended dividing the competencies of nurses into ten categories: health promotion and disease prevention, treatment, palliative care, ethics and regulation, assessment and taking action, covering basic needs, communication and documentation, responsibility and activeness, cooperation and attitudes towards older people (Bing‐Jonsson et al., 2016).

*The nursing process* is an important and appropriate method to describe and explain the core of nursing concerning its scientific base and practice. Inherent in this view are values, humanistic assumptions and critical thinking in the decision‐making process (Hagos et al., 2014). Hagos et al. (2014) argue that as nurses comprise the largest group of health professionals, they impact the overall effectiveness of the healthcare service. Following this argument, the nursing process plays an important role in achieving high‐quality healthcare services (Hagos et al., 2014). How nurses effectively solve complex clinical problems in various contexts is dependent on their ability to apply their skills (Vatnøy et al., 2019). This capability relies on pattern recognition, which identifies changes in the patients` clinical situations. This ability is also crucial to nursing and clinical decision‐making processes (Banning, 2008) and is dependent on having knowledge of and experience in a specific area of nursing. However, the skill of pattern recognition relies on memory, which may even lead nurses to misunderstand or inadequately evaluate a clinical situation (Banning, 2008). Therefore, nurses risk making mistakes if they rely only on their clinical skills without any support from clinical screening tools (Vatnøy et al., 2019).

*Clinical screening tools* are important in assessing a patient's clinical status, changes to clinical status and evaluation of clinical action steps, but the use should be combined with advanced clinical competency. During the process of clinical reasoning and evaluation, situated knowledge—not merely the acquisition of knowledge—must be used. Quite simply, if they have a poor or incorrect understanding of their patient's situation, they will not be able to address their patients’ needs (Burgers et al., 2020). The use and potential benefits of clinical screening tools are dependent on clinical competence and knowledge (Downey et al., 2017). Understanding the assessment decisions of community nurses when providing care is vital. Increased knowledge about nurses’ perceptions concerning examination and clinical screening tools may contribute to improved nursing practice in the community healthcare sector.

Understanding Registered Nurses’ (RNs) perceptions and addressing their questions about the clinical examination and screening tools—whose purpose is to ensure patient safety and quality of care—will enlighten practice. This study describes the examination practices and clinical evaluation processes that RNs perform in the community healthcare sector.

### 2.1 Research questions

1. How are Registered Nurses (RNs) performing clinical assessment of patients in daily work in the community healthcare sector?
2. What are RNs’ perceptions of their examination practices and clinical evaluation processes in the community healthcare sector?

## 3 THE STUDY

### 3.1 Design

A qualitative exploratory design was used to gain an in‐depth understanding of RNs’ perceptions of their daily examination practices and clinical evaluation processes in the community healthcare sector. Descriptive design is chosen to develop a rich understanding of the experiences and meaning of an individual related to a phenomenon (Bradshaw et al., 2017).

### 3.2 Methods

#### 3.2.1 Sample and context

This study was conducted in the Norwegian community healthcare sector,and the convenience sampling method guiding this sample recruitment process is congruent with the guidelines in the literature (Polit & Beck, 2020). Community‐based healthcare services are characterized by professional healthcare teams that work in patients’ homes. The local directors in fifteen municipalities in southern Norway were contacted by email and asked whether they would be interested in recruiting RNs to participate in an interview study. The local directors approached the RNs, and the contact information of those who agreed to participate was conveyed to the authors. The potential participants were contacted, and convenient interview dates and venues were arranged. Participation criteria required RNs to be employed in a relevant community healthcare sector and have more than one year of work experience. Three municipalities were not able to participate in the study because of their extreme workloads at the time. A total of 20 RNs (18 females and two males) working in twelve municipalities in Norway were individually interviewed from August 2017 to March 2018. The participants’ ages ranged from 28–61 years (mean = 43.7 years), and they had worked 2–26 years in the municipality (mean 11.2 years). Three participants specialized in palliative care, four in management, five in geriatric care, two in academic guidance and one had a master's degree in clinical health sciences (see Table 1).

**TABLE 1:** Demographic characteristics of the Registered Nurses interviewed (*N* = 20)

Characteristics	Number (per cent)
Gender	
Female	18 (90)
Male	2 (10)
Age (years)	
20–25	0
26–−30	1 (5)
31–35	3 (15)
36–40	5 (25)
41–45	3 (15)
46–50	3 (15)
51–above years	5 (25)
Highest qualification completed	
Bachelor's degree	19 (95)
Master's degree	1 (5)
Number of years working as a Registered Nurse (years)	
2–5	1 (5)
6–10	7 (35)
11–20	8 (40)
21–30	4 (20)
Number of years working in a hospital	
Mean, min/max	2.4 0/10
Number of years working in primary Care	
Mean, min/max	11.8 2/26
Number of years at the current ward	
Mean, min/max	11.2 2/26

#### 3.2.2 Interview guide

A semi‐structured interview guide was developed for the study and consisted of eight broad questions related to participants’ daily work situations in community health care. The interview questions were developed based on earlier literature (Bing‐Jonsson et al., 2016; Downey et al., 2017) and designed to elicit participants’ views on their daily work, including examination practices and clinical evaluation processes.

#### 3.2.3 Procedure

Individual interviews were conducted at the community healthcare offices in a room chosen by the participants; interviews lasted 13–52 min (mean = 24.1 min).

The interviews were conducted by the authors, recorded and transcribed verbatim. The researchers were all experienced in qualitative methods (including conducting interviews) and did not know the participants.

### 3.3 Analysis

The analysis was conducted in fall 2018. The transcribed interviews were analysed in six steps using Braun and Clarke (2006) thematic analysis. In the first step, one of the authors uploaded the interview transcript in Nvivo 12 (QSR, 2012), and all the authors read the interview transcripts while actively searching for meanings and patterns in them. Any patterns found were then discussed among the authors. In the second step, one of the authors (KJ) used Nvivo to generate the highest number of possible initial codes. These were then cross‐checked by two other authors (ÅS, MF). In the third step, all authors searched for themes based on the initial codes. In the fourth step, all the themes were reviewed, and in the fifth step, the themes were defined and named. Finally, the themes were reported based on sufficient evidence from the data.

### 3.4 Ethics

The study was approved for correct data storage and handling by the Norwegian Centre for Research Data (NSD), a national committee acknowledged by the municipalities. Participants were informed through written and oral communication that their participation in the research was voluntary, and they could withdraw their consent at any time before the data were analysed. Their confidentiality was ensured throughout all phases of the study. Transcripts were de‐identified to ensure confidentiality.

## 4 RESULTS

We found four themes: (1) examinations and evaluations are embedded in an RN’s daily work, (2) significance of an RN’s clinical competency, (3) different tasks require various roles and (4) access to resources and equipment.

### 4.1 Examinations and evaluations are embedded in an RN’s daily work

The examination and evaluation conducted by RNs in their daily work were often connected to other aspects. However, the role of clinical observations is a key theme. In addition, there were three sub‐themes about which evaluations were undertaken: (1) when receiving a new patient, (2) when providing daily care and (3) when performing systematic evaluations. Whenever a new patient was admitted to home healthcare services or a nursing home, there were several evaluations made by RNs, who assessed patients’ medical, practical and social information. Examples of evaluations included blood pressure, pulse, temperature and weight. In addition, it is important to evaluate the patients’ personal data and practical issues. Although RNs could inform nursing assistants (NAs) about their observations, sometimes they needed to make observations as they had the skills necessary for taking care of the patients. It was necessary to spend time with the patients to assess their condition. They evaluated whether the patient was coherent and could converse normally or whether they were repeating themselves because they were unable to make themselves understood. This determination could not be made after a brief conversation. In contrast, the process was time‐consuming, or, as one RN stated:

Throughout the visit, we observe patients’ skin colour, temperature, breathing, movements, if there is anything wrong and, if so, if it is something psychological … If they are depressed, whether this is a feeling that will last a while. (Interview 5)

It was important for these RNs to see the whole person from a physical, psychological and social standpoint. Their nursing skills were based on experience and education and were necessary for them to observe any changes in their patients’ condition. These observations were based on internalized nursing skills, which helped them know what signs to look for.

You go in to the patient, you say “good morning.” They have something to drink. You ask how their night has gone. If the patient is bedridden then it’s natural to examine their whole body, help them to sit upright in bed if they can, and if they do sit up, examine their back as well … Whether their skin is dry and whether their legs need to be moisturized. (Interview 14)

However, as shown in the next sub‐theme, in addition to these informal examinations, there were several lists and questionnaires that the RNs had to follow to make more systematic evaluations. For instance, there were questionnaires and lists for evaluation of patients’ conditions when they were admitted to either home health care or a nursing home, or when their condition changed over time. There were also checklists covering patients’ diets, medications, vital signs, social networks and general functions. The RNs assessed whether patients experienced a drop in their physical or psychological functions.

Because [the evaluation] is then in relation to patients’ medications, circulation/breathing, skin tone, mucous membranes, diet, teeth, mouth, digestion, mobility, pain level, vision, hearing, mental/cognitive function, sleep, housing, and medical equipment. (...) There is also risk assessment, so you should think a bit about different risk areas. (Interview 10)

Additionally, the patient's social situation was assessed by RNs, especially for patients with dementia living at home. Some patients lived alone and had no relatives to help them, while others had a spouse or child who could assist them at home. It was important for the participants to know about patients’ social situations, as this was a starting point that they used to determine the type and amount of help each patient would receive.

### 4.2 Significance of the RN’s clinical competency

Clinical competency was based on two main components: education and clinical experience. While it was important for RNs to have experience in assessing the patient's condition, an evaluation tool could help them to define a change in their patients’ condition. One RN explained the need to have a combination of tacit knowledge and evaluation tools.

It's that tacit knowledge that you can… And so, you enter a room and think that something has happened. However, this cannot be described. So, you think it might be okay to run a MEWS assessment. (Interview 7)

This tacit knowledge was a result of experience combined with theoretical knowledge from training as an RN or specialist nurse, for instance in oncology or dementia. *Reflection‐in‐action* and *reflection‐on‐action* processes were learned after graduation from nursing school through continuing education programmes. The combination of education and experience after clinical treatment of numerous patients over a long period gave RNs a high level of clinical competency, which increased their capabilities for making high‐quality evaluations. Their tacit knowledge often provided an intuition that their patient's condition had deteriorated, and when this knowledge was combined with systematic evaluation tools, RNs could provide their patients with accurate evaluations that helped them to cope with difficult situations.

### 4.3 Different tasks require various roles

This sub‐theme concerns the situations in which RNs organized their daily tasks. There were five topics to be considered. One of these was work schedules, which divided daily tasks among staff members to ensure that all tasks were completed during the workday. The RNs performed different tasks each day depending on the day's work schedule. On certain days, they were group leaders and completed several systematic evaluations; on other days, they remained with patients making observations during care administration. Another aspect of this sub‐theme was the overall organization of the healthcare system in the request‐and‐provider model, where one office alone decides the amount of healthcare service individual patients would receive, and then, the home health services provide this care. This type of organization had implications for how patients’ needs were met by RNs. There were different tasks involved, such as ordering medication at the pharmacy, with associated documentation as well as issues about technology and electronic health records (EHR) systems. Although not all the EHRs were adapted to the tasks or evaluations the RNs had to make, an important aspect of this task was providing care based on trust and building trust with patients who were not used to receiving help for their personal care needs. The following quotation exemplifies this approach to meeting patients’ needs:

We listen to the patient. This is perhaps one of the most important parts of caregiving: listening to and seeing the patient. (Interview 9)

This theme concerned first, the overall organization of community healthcare services; second, specific organizations about technology and EHR; and third, the human aspect of meeting patients with a holistic attitude. This theme also included the priorities set by the healthcare services and the professional reasons used to determine the provision of care for individual patients, the context for this care and how caring roles were established and conducted. Professional reasoning was also relevant for RNs when assessing the results of their evaluations. As one RN explained:

You learn through the work. In this profession, you never stop learning. This is about understanding. If you do not understand what you have observed, you cannot do the right thing [for your patients]. (Interview 14)

The various community healthcare services raised different arguments about who was responsible for the initial evaluation when patients returned from the hospital. For instance, in one community, the service had a checklist for what should be observed and done when the patient came home, ensuring that every patient received the same systematic care. However, in another community, there was one RN assigned to completing the checklists for all their patients to maintain consistency and ensure that everyone received the same quality of care.

The participants discussed the fact that evaluations differed according to the context. For example, some nursing homes specialized in treating people with dementia, while others offered short‐term places where residents stayed for a time and then returned home. Additionally, home healthcare participants had different ways of prioritizing care depending on their primary nursing care or worklist organization. There were also discrepancies about the participants’ views on the different roles in the healthcare system. Some participants emphasized the unique tasks performed by the RNs, so there were specialist nurses who took care of such diverse areas as wounds, diabetes, dementia and cancer. Finally, in some communities, nursing assistants could undertake screenings, even though RNs always contacted physicians whenever patients were deteriorating.

### 4.4 Collaboration and access to resources and equipment

Participants described how they collaborated with their rehabilitation teams, all of which were interdisciplinary. This cooperation and collaboration comprised one sub‐theme for the participants about the clinical evaluations of patients’ conditions. Indeed, interdisciplinary collaboration was an important part of the systematic evaluation process, as collaboration took place among the rehabilitation team, cancer coordinator (who was often a nurse), dementia team, general practitioner (GP), occupational therapist and physiotherapist.

Rehabilitation teams are also very good for evaluation. We can report that a patient shows a drop in functioning levels, and then they can make a very thorough evaluation to see why this is happening. Because it is interdisciplinary, there is a nurse and physiotherapist as well as a skilled healthcare assistant on this team. (Interview 1)

Although these team members had different roles and tasks, they knew about each other's competency areas and said that interdisciplinary collaboration was important for making both evaluations and interventions to meet their patients’ different needs.

Access to equipment was another resource that enabled the participants to make high‐quality evaluations. Several of them described an emergency bag they had recently been provided with when visiting patients in their homes:

However, we also have emergency bags that contain a blood pressure gauge, a thermometer, an oxygen meter, urine test strips, skin and wound care supplies, infectious disease equipment, and the kind of mask you blow into in case of cardiac arrest. (Interview 10)

Although RNs had access to all the equipment and data they needed to work, they were not always used properly—or used at all. Resources such as the provision of adequate time and training for correct use of their equipment were appreciated by participants but were sometimes lacking. Nonetheless, most participants found that they did have many of the resources required to make the evaluations necessary for providing proper patient care.

## 5 DISCUSSION

A detailed discussion of the four themes indicates how the RNs performed clinical assessments of patients in their daily work and what their perceptions of examination practices and clinical evaluation processes in the community healthcare sector were. This information begins to highlight the answers to our research questions.

This study gathered knowledge about how RNs complete and experience their examination practices and clinical evaluation processes in the community healthcare sector. One of our main findings was that examinations and evaluations were embedded in RNs’ daily work. The RNs reported that they completed several observations during their regular caregiving activities, observing patients during their morning caregiving sessions, or through having in‐depth conversations during their caregiving activities. An RN’s clinical knowledge is gained through their experiences with many patients in similar situations, and the RNs in our study said that they made observations systematically both by observing clinically and using systematic checklists for evaluating their patients’ situations. RNs’ decision‐making may be influenced by education, attitude and experiences (Banning, 2008). To ensure accurate observations and provision of care for vulnerable patients, checklists should be customized to examine and evaluate each patient's needs in a systematic manner (Nilsen et al., 2019).

We found that the RNs’ clinical competency was of great importance when using checklists in the community healthcare sector. This is congruent with the findings of Nilsen et al. (2019), who emphasize that RNs should have versatile and extensive skills to use checklists appropriately. The need for advanced assessment skills in the community healthcare sector has also been highlighted in several other studies (Raleigh & Allan, 2017; Vatnøy et al., 2019). Head administrators must ensure that their staff members have the skills required to use different checklists, according to Nilsen et al. (2019). As St John and Keleher (2020) state, healthcare staff members’ competence must be based on broad theoretical knowledge from nursing and other disciplines. In addition, these staff members must use their knowledge in clinical situations according to their patient's current status (St John & Keleher, 2020). According to our findings, these factors must be considered when using checklists, as other healthcare staff members also use checklists. These require different skills to provide appropriate answers about patients’ conditions. Cooperation between different experts is necessary to provide adequate nursing care. This was confirmed in the study by Bing‐Jonsson et al. (2016), who found ten different competencies that RNs required. Professional group affiliation, workplace and age were found to influence competency levels in community health care (Bing‐Jonsson et al., 2016). We also found that the various functions performed by an RN in their unit required the completion of different tasks using different competencies.

We found that RNs had different foci during their workday depending on the worklists assigned to them, including descriptions of the necessary tasks to be performed among different patients. RNs were dependent on having necessary equipment and assistance and were mostly satisfied with the equipment provided by their community healthcare services. However, some of the RNs stated that being provided with their own bags containing necessary equipment was helpful. We did not find that equipment and assistance were statistically significant factors in any of our other research projects; however, in this study, RNs emphasized this as a requirement for effective delivery. The RNs said that they have their own equipment bags, so it is their responsibility to calibrate and ensure that their equipment is functioning correctly at any given time.

The implementation of early warning scoring systems such as Modified Early Warning Score (MEWS) in clinical practice to enhance the quality of care has not been documented (Bedoya et al., 2019). It is a requirement that MEWS is applied thoroughly; at the same time, this application should be combined with common sense and professional judgment, or *phronesis*. Clinical observation of the patient is required in combination with MEWS (Downey et al., 2017). In this manner, MEWS could be an appropriate tool for helping RNs communicate with physicians and observe any deterioration in their patients’ conditions. RNs must systematically make conscious evaluations of their patients’ conditions (Downey et al., 2017). The working environment of healthcare units might improve whether screening tools such as MEWS are used properly. A high level of nursing competency is needed to research information when there are indicators that something is wrong with a patient (St John & Keleher, 2020). There has been debate about whether RNs can be trusted to complete the scores accurately, or whether there are a number of RNs who have not been trained well enough to apply MEWS systematically (Downey et al., 2017). Systematic training may have a positive impact on competency levels; however, this might also be contradictory in some cases (Jensen et al., 2018). Healthcare professionals and administrators in the healthcare sector should be aware of this challenge.

We found that organizational factors were important for using checklists and instruments when evaluating patients’ conditions. The RNs were loyal to both their professional and organizational values concerning implementing new checklists and systems in the healthcare sector. Professionals must follow the guidelines and procedures that are standard for community healthcare services in which they are working (Nilsen et al., 2019).

### 5.1 Limitations

This study meets the criteria for trustworthiness, which includes confirmability, credibility, dependability and transferability (Schwandt et al., 2007). The study recruited RNs from different communities, home healthcare services and nursing homes. This is congruent with the criteria of confirmability and credibility. The researchers are familiar with the professional context, as they are all trained RNs and professors in the nursing field. They are experienced with interviewing as a research method and meet the requirements for dependability. The researchers included four female and one male RNs, of whom two have a master's degree and three have a Ph.D. in nursing. The analysis was conducted by everyone on the research team. The analysis was discussed until agreement on the themes was achieved. The context and research process are described thoroughly, so transferability to similar contexts should be possible, and thus, the requirement for trustworthiness is met.

### 5.2 Clinical relevance

This study indicates that RNs in the community healthcare sector should use proper examination tools combined with evidence‐based knowledge to evaluate their patients’ conditions (and any deterioration). The significance of RNs receiving support from other team members was emphasized. Future work is needed to identify what RNs may need by way of further education and training to complete high‐quality evaluations. In addition, research on professional values and staff members’ understanding of and loyalty to appropriately using clinical screening tools is recommended.

## 6 CONCLUSIONS

Findings from this study show that RNs have competencies that fall in a range of different examination practices and clinical evaluation processes in the community healthcare sector. They perceive their practice as complex, perform evaluations in their daily work, and rely on other healthcare professionals, facilities, and equipment to provide patients with high‐quality care.

## CONFLICT OF INTEREST

No conflict of interest has been declared by the authors.

## AUTHOR CONTRIBUTION

All authors contributed in designing and conducting the study, in the analytic process and drafting the first version of the manuscript, and all authors reviewed, edited and approved the final version. The corresponding author attest sthat all authors meet the authorship criteria.

## Data Availability

The data that support the findings of this study are available on request from the corresponding author. The data are not publicly available due to privacy or ethical restrictions.
